# A comparison of multiband and multiband multiecho gradient‐echo EPI for task fMRI at 3 T

**DOI:** 10.1002/hbm.26081

**Published:** 2022-10-05

**Authors:** Zahra Fazal, Daniel E. P. Gomez, Alberto Llera, José P. R. F. Marques, Thomas Beck, Benedikt A. Poser, David G. Norris

**Affiliations:** ^1^ Donders Institute for Brain, Cognition and Behaviour, Donders Centre for Cognitive Neuroimaging Radboud University Nijmegen Nijmegen The Netherlands; ^2^ Athinoula A. Martinos Center for Biomedical Imaging Massachusetts General Hospital Boston Massachusetts USA; ^3^ Siemens Medical Solutions Erlangen Germany; ^4^ Faculty of Psychology and Neuroscience Maastricht University Maastricht Netherlands; ^5^ Erwin L. Hahn Institute for Magnetic Resonance Imaging, UNESCO‐Weltkulturerbe Zollverein, Leitstand Kokerei Zollverein Essen Germany; ^6^ Present address: Department of Biomedical Engineering Boston University Boston Massachusetts USA

**Keywords:** AROMA, FIX, fMRI, multiband, multiecho

## Abstract

A multiband (MB) echo‐planar imaging (EPI) sequence is compared to a multiband multiecho (MBME) EPI protocol to investigate differences in sensitivity for task functional magnetic resonance imaging (fMRI) at 3 T. Multiecho sampling improves sensitivity in areas where single‐echo‐EPI suffers from dropouts. However, It requires in‐plane acceleration to reduce the echo train length, limiting the slice acceleration factor and the temporal and spatial resolution Data were acquired for both protocols in two sessions 24 h apart using an adapted color‐word interference Stroop task. Besides protocol comparison statistically, we performed test–retest reliability across sessions for different protocols and denoising methods. We evaluated the sensitivity of two different echo‐combination strategies for MBME‐EPI. We examined the performance of three different data denoising approaches: “Standard,” “AROMA,” and “FIX” for MB and MBME, and assessed whether a specific method is preferable. We consider using an appropriate autoregressive model order within the general linear model framework to correct TR differences between the protocols. The comparison between protocols and denoising methods showed at group level significantly higher mean *z*‐scores and the number of active voxels for MBME in the motor, subcortical and medial frontal cortices. When comparing different echo combinations, our results suggest that a contrast‐to‐noise ratio weighted echo combination improves sensitivity in MBME compared to simple echo‐summation. This study indicates that MBME can be a preferred protocol in task fMRI at spatial resolution (≥2 mm), primarily in medial prefrontal and subcortical areas.

## Graphical abstract

In summary, we found that multiband multiecho (MBME) and MB give a comparable performance in most brain regions with this comparison study. In the regions with dropouts and susceptibility induced inhomogeneity, MBME performed somewhat better at the group level. The results showed that the difference between sequences is more significant than between different denoising (FIX and AROMA) strategies at the group level, highlighting the importance of considering an optimized sequence before data collection. For MBME, choosing an optimal echo combination can influence the sensitivity at the group‐level analysis.

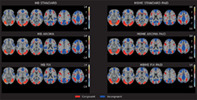

## 1 INTRODUCTION

Gradient‐echo echo‐planar imaging (GE‐EPI; Mansfield, 1977) is the most commonly used sequence in functional magnetic resonance imaging (fMRI) (Bandettini et al., 1992; Kwong et al., 1992; Ogawa et al., 1990) due to its high sensitivity to blood oxygenation level dependent (BOLD) contrast and fast acquisition speed. Typically, the acquisition time is several seconds for whole‐brain coverage. Using parallel imaging (PI; Griswold et al. 2002a; Pruessmann et al., 1999; Sodickson & Manning, 1997) echo train length (ETL) can be reduced considerably, which allows for a higher spatial resolution to be practically achievable and reduces distortion artifacts. However, in‐plane acceleration does not translate to a corresponding TR reduction by the acceleration factor in fMRI, where a fixed TE is necessary for optimum BOLD contrast (at TE ~ T2*) (Menon et al., 1993). On the contrary, simultaneous multislice (SMS) or multiband (MB) (Larkman et al., 2001; Nunes et al., 2006) techniques allow for a significant increase in temporal resolution by exciting and acquiring multiple slices simultaneously. Thereby reducing acquisition time by a factor N without decreasing echo time and without a substantial signal‐to‐noise (SNR) penalty. MB can however increase SAR and RF peak power (which may require lower bandwidth RF pulses) and gives a reduced steady‐state signal due to shorter TR. The development of robust reconstruction techniques (Moeller et al., 2010) and the invention of blipped CAIPIRINHA (Setsompop et al., 2012) brought the benefits of CAIPIRINHA (Breuer et al., 2006) to EPI. All these factors contributed to the widespread implementation and use of SMS techniques, including in the human connectome project (HCP; Feinberg et al., 2010; Uğurbil et al., 2013).

MB‐EPI has become the method of choice for acquiring BOLD fMRI data (Feinberg et al., 2010; Uğurbil et al., 2013), effectively replacing standard single‐band 2D‐EPI. Despite being commonly used, the benefits of a higher temporal resolution are not readily apparent due to the slow BOLD hemodynamic response. Nevertheless, the higher sampling rate in MB makes it possible to adequately sample the respiratory and, to some degree, cardiac frequencies, thus increasing temporal SNR (tSNR) (Griffanti et al., 2014) by allowing the efficient removal of physiological noise from the data. A faster sampling rate with shorter TR increases the number of volumes acquired per unit time, thereby improving the statistical outcome in task fMRI compared to single‐band (Demetriou et al., 2018; Todd et al., 2017, Todd et al., 2016; Chen et al., 2015). However, to rigorously assess the sensitivity gain at higher MB factors, the evaluation needs to consider that the data sets have different sampling rates, numbers of volumes, and temporal autocorrelation. When comparing the various software packages available (Olszowy et al., ) recommended using SPM (FAST) or AFNI (Analysis of Functional NeuroImages) for short‐TR data, to obtain an unbiased estimate of statistical values.

The attractiveness of ME acquisitions over single echo lies in compensation for variations in T2*(Speck and Hennig, 1998, Hagberg et al., 2002). In ME‐EPI, images are acquired with different echo times after a single RF excitation. Acquiring signals at multiple TEs makes it possible to increase the BOLD contrast‐to‐noise ratio (CNR) across the brain by combining images from different echo times (Posse et al., 1999). In comparison to standard single‐echo EPI, ME‐EPI combined with PI showed an increase in functional sensitivity at both 3 T (Poser et al., 2006) and 7 T (Poser & Norris, 2009) with considerable gains seen in the regions suffering from susceptibility induced inhomogeneities. The drawback of combining ME with MB is that ME usually requires parallel imaging to shorten the ETL, limiting the maximum MB acceleration possible for an multiband multiecho (MBME) protocol, because in‐plane (GRAPPA/SENSE) (de Zwart et al., 2002) and through‐plane (SMS) parallel imaging techniques have to share the same total under‐sampling power. Nonetheless, the benefits of MBME relative to ME‐EPI have previously been demonstrated for resting and task fMRI with Stroop task by (Boyacioğlu et al., 2015) in resting fMRI (Olafsson et al., 2015). A recent comparison of MBME with MB, in task fMRI with visual checkerboard task, showed high activation volume and high sensitivity for MBME than MB (Cohen et al., 2021).

With recent advances in MR sequences, new data‐driven approaches have been developed for denoising the data (Griffanti et al., 2014; Pruim et al., 2015; Salimi‐Khorshidi et al., 2014). A widely used data‐driven method is FMRIB's ICA‐based X‐noiseifier (ICA‐FIX) (Griffanti et al., 2014) for removing motion and physiological artifacts. ICA‐FIX was explicitly designed to denoise the MB accelerated resting‐state fMRI data in the HCP, and (Boyacioğlu et al., 2015) were early users of ICA‐FIX for denoising task‐fMRI data. An alternative ICA‐based method that requires no training is the ICA‐based automatic removal of motion artifacts (ICA‐AROMA; Pruim et al., 2015). Previous work has shown that ICA‐AROMA and ICA‐FIX improve resting‐state network reproducibility and reduce loss in temporal degrees of freedom compared to spike regression and scrubbing (Pruim et al., 2014), as well as enhancing tSNR (Griffeth et al., 2013).

In the present study, we examine whether MBME offers an advantage over MB when acquired and analyzed according to current standard practices in fMRI. The data were obtained using a color‐word matching interference Stroop task, known to induce widespread activity throughout the brain, including regions of low static field homogeneity (Boyacioğlu et al., 2015; Norris et al., 2002; Poser & Norris, 2009). The main objective of this study was (i) to examine the sensitivity difference between MBME and MB within the activated regions. (ii) To evaluate the test–retest reliability of both protocols. (iii) To determine how different echo combination methods can improve the sensitivity of MBME and, (iv) to compare three different denoising strategies: STANDARD (removal of motion and use of physiological noise regressors), FSL's FIX, and AROMA across protocols to determine the preferred denoising method.

## 2 METHODS

### 2.1 Data acquisition

In this study, 14 healthy volunteers (3M/11F, 21.6 ± 2.0 years old) with normal to corrected vision participated. All participants provided written informed consent according to the local ethics committee (Committee on Research Involving Human Subjects, region Arnhem‐Nijmegen, The Netherlands). Each participant was scanned twice for data acquisition for 30 min in two sessions, 24 h apart. Functional data sets were acquired twice in counterbalanced order within each session with MB and MBME protocols to avoid possible confounding habituation effects during analysis. The functional data set consisted of an 8‐min color‐word matching Stroop interference task (Zysset et al., 2001). During the scanning sessions, participants lay supine in the MRI scanner.

### 2.2 Scanning protocols

Preliminary experiments from Gomez et al. (2015) determined the choice of protocols. MBME requires a substantial reduction of the ETL, achieved by increasing the readout bandwidth and utilizing PI. In contrast, MB protocols benefit from lower bandwidths, which reduce noise. Due to such trade‐offs, we optimized both protocols separately to have fast TR with full brain coverage at a spatial resolution of 2.5 mm isotropic without reducing efficiency. The flip angle for each protocol was the Ernst angle based on the TR values and approximate gray matter T1 of 1200 ms. All data were acquired on a 3 T Siemens MAGNETOM Prisma MRI scanner (Siemens Healthineers) with a 32‐channel head coil, using an MBME GE‐EPI sequence provided as a WIP from Siemens Healthcare.

Both MB and MBME acquisition protocols had the following parameters in common: in‐plane resolution 2.5 mm isotropic and 48 slices without a gap for a coverage of 120 mm, FOV 210 × 210 mm^2^, a blipped‐CAIPI shift of 1/3 FOV (Setsompop et al., 2012), and fat saturation performed before each RF excitation. The parameters specific to each protocol are reported in Table 1.

**TABLE 1:** EPI protocol parameters

	MB factor	GRAPPA factor	TEs (ms)	TR (ms)	rBW (Hz/px)	FA	Number of volumes	PF
MB only	6	1	38	584	1985	56	2011	1
MBME	3	2	15,36,54	1260	2205	69	926	7/8

Abbreviations: EPI, echo‐planar imaging; FA, flip angle; GRAPPA factor, in‐plane acceleration factor; MB factor, slice acceleration factor; MBME, multiband multiecho; PF, partial Fourier; rBW, readout bandwidth; TEs, echo times; TR, repetition time.

The total acceleration factor was the same for both protocols. Anatomical scans were acquired for image registration using a sagittal 1 mm isotropic MP‐RAGE with a TR of 2300 ms, a TI of 900 ms, a TE of 3 ms, a flip angle of 9°, a turbo factor of 16, and an in‐plane acceleration factor of 2 with a total acquisition time of 5 min and 12 s. All imaging sequences were automatically aligned using an auto‐align localizer sequence.

For MBME, a Partial Fourier of 7/8 was utilized reconstructed using standard Siemens reconstruction. This performs zero filling, thereby slightly increasing the SNR. However, as we are in the physiological regime (Triantafyllou et al., 2005), the SNR gain does not increase the tSNR in the fMRI time series. Images were reconstructed online using Slice‐GRAPPA (Setsompop et al., 2012) with LeakBlock (Cauley et al., 2014). For in‐plane acceleration, only used in the MBME protocol, ACS reference lines GRAPPA calibration were acquired using FLEET (Polimeni et al., 2016). Single slice reference k‐space for slice‐GRAPPA calibration was obtained at the shortest TE to avoid the introduction of additional variance.

### 2.3 Activation study

An adapted version of the color‐word matching Stroop interference task has been utilized as previously described in detail (Zysset et al., 2001). This experimental design was employed previously (Boyacioğlu et al., 2015; Poser et al., 2006; Poser & Norris, 2009) to compare protocols and provided reproducible group activation maps with relatively small sample sizes across different studies, indicating it as a good choice for comparing protocols with different sensitivity.

**FIGURE 1:**
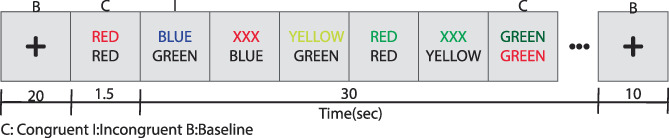
An adapted version of the color‐word matching Stroop task. The two conditions used for the task are congruent (C) and incongruent (I). Participants had to decide in each condition: “Does the color of the upper row word correspond with the meaning of the word written below in black?” the correct responses would be from the congruent trials, from the first and the last trial. The responses are considered incorrect if provided at incongruent trials.

This task also induces widespread activation across the brain, making it a suitable choice for comparing different protocols in terms of sensitivity across different brain regions (Boyacioğlu et al., 2015; Norris et al., 2002; Poser & Norris, 2009). The Stroop task elicits activity in visual, motor, and frontal areas, such as the cingulate cortex and medial prefrontal cortex (Zysset et al., 2001), in the parietal lobe, parietal sulcus, and dorsal visual stream, and focal activity in subcortical areas (Saban et al., 2018). The Stroop task used here is illustrated in Figure 1. During the baseline condition, a black cross was shown on the screen. During the activation condition, two‐color words were displayed over each other. Participants were asked to press a button only in the congruent condition, that is, when the bottom word corresponded to the upper word's color. Trials, where this correspondence was not correct were called incongruent trials. The activation condition consisted of twenty 1.15‐s intermixed congruent and incongruent trials, totaling 23‐s per block. Responses were considered correct if provided in the congruent trials and incorrect if given for incongruent trials. There was a 20‐s resting baseline at the beginning of the task. All subsequent baseline blocks lasted for 10 s. The total duration of the task was 8 min for each protocol, 80‐s resting‐state scans for MBME to calculate tSNR in parallel‐acquired inhomogeneity‐desensitized (PAID) weighting followed by a 6 min T1 weighted image (~30 min scanning). Stimuli were presented, and button presses were recorded using presentation (Neurobehavioral Systems Inc.). Before performing the task, participants were instructed on a desktop computer next to the scanning console to guarantee that they understood the procedure.

### 2.4 Data preprocessing

Before data preprocessing, DICOMs were converted to NIfTI's and organized according to the Brain Imaging Data Structure (Gorgolewski et al., 2016) using dcm2niix (Li et al., 2016). Data from both the MB and MBME protocols were preprocessed using fMRIprep v1.0.8 (Esteban et al., 2018), based on Nipype (Gorgolewski et al., 2011).

### 2.5 Functional processing

For each BOLD fMRI time series (both MB and MBME), the following preprocessing was performed using fMRIPrep v1.0.8 (Esteban et al., 2018). First, a reference volume and its skull‐stripped version were generated. A deformation field to correct for susceptibility distortions was estimated based on fMRIPrep's field map‐less approach. The deformation field is estimated by co‐registering BOLD reference data to the same‐subject intesting inverted T1w‐reference maps (Huntenburg,  Wang et al., 2017). Data registration is performed with antsRegistration (ANTs 2.2.0). The process is regularized by constraining deformation to be nonzero only along the phase‐encoding direction and modulated with an average field map template (Treiber et al., 2016). An unwarped BOLD reference was calculated for a more accurate co‐registration with an anatomical reference based on the estimated susceptibility distortion. Head‐motion parameters for the BOLD reference (transformation matrices and six corresponding rotation and translation parameters) were estimated using MCFLIRT (FSL 5.0.11; Jenkinson et al., 2002). The BOLD time‐series were resampled onto their original, native space by applying a single, composite transform to correct head‐motion and susceptibility distortions. These resampled BOLD time‐series will be referred to as preprocessed BOLD. The BOLD reference was then co‐registered to the T1w reference using FLIRT (FSL 5.0.1, Jenkinson & Smith, 2001) with the boundary‐based registration (Greve & Fischl, 2009) cost‐function. Co‐registration was configured with nine degrees of freedom to account for distortions remaining in the BOLD reference. The BOLD time‐series were resampled to MNI152NLin2009cAsym standard space, generating a preprocessed BOLD run in MNI152NLin2009cAsym space. All resampling was performed with a single interpolation step by composing all the pertinent transformations (i.e., head‐motion transform matrices, susceptibility distortion correction when available, and co‐registration to anatomical and template spaces). Gridded (volumetric) resampling was performed using antsApplyTransforms (ANTs), configured with Lanczos interpolation to minimize the smoothing effects of other kernels. Finally, data sets were smoothed with a 5 mm kernel and high pass filtered with a cut‐off frequency of 1/100 s.

MBME data sets were combined with two different echo combination methods: All these methods were based on image‐by‐image estimation.

1. Using averaging for combining the echoes.
2. Combining according to their CNR; a combination herein referred to as PAID weighting, described in Equation (1) (Poser et al., 2006).

(1) $$W_{i}=\frac{{\mathrm{SNR}}_{i}{\mathrm{TE}}_{i}}{\sum {\mathrm{SNR}}_{i}{\mathrm{TE}}_{i}}.$$

### 2.6 Data denoising

Three different data denoising strategies were used for both MB and MBME, which we describe as “Standard,” “FIX” (Griffeth et al., 2013), and “AROMA” (Pruim et al., 2015) denoising. In the next subsections, we briefly detail each of these denoising approaches.

### 2.7 Standard denoising

In “standard denoising,” six motion parameters (three translation, three rotation), cerebrospinal fluid (CSF), and white matter signals were regressed out of the preprocessed smoothed data as estimated with aCompCor in fMRIprep v1.0.8 (Esteban et al., 2018). Spatial smoothing was performed with a 5 × 5 × 5 mm^3^ full width at half maximum using SUSAN (Smith & Brady, 1997), before performing the general linear model (GLM) in SPM (Friston et al., 1994). The smoothing order was kept consistent for making this pipeline comparable to the FIX and AROMA (see the following two subsections). In MBME, the motion regressors were calculated from the first echo. The parameters from the motion correction are retained and applied across all echoes. Finally, echoes were combined using a PAID weighting see Equation (1).

### 2.8 FIX denoising

Using the same scanning protocols and procedures described earlier in Section 2.1, we acquired task data sets from 10 additional healthy subjects and used them to train the FIX classifier (Griffanti et al., 2014; Salimi‐Khorshidi et al., 2014) The FIX training procedure consisted of applying spatial ICA with a dimensionality of 70 on each of the 10 training data sets and manually labeling components as either signal or noise following recommendations from the literature (Griffanti et al., 2017). This classifier was subsequently used to denoise the primary data considered in this paper. To that end, each experimental fMRI data set was decomposed into 70 independent components and classified as signal or noise using the pretrained FIX classifier. Components identified as noise were regressed out of the original data (Griffanti et al., 2014).

We followed standard practices of training the FIX classifier on MBME data (Dipasquale et al., 2017): multiple echoes were first combined with different weighting schemes and later decomposed into IC components. We trained the FIX classifier, in MBME data, by combing multiple echoes using simple averaging. However, before merging the echoes, the motion parameters were estimated from the first echo because of high SNR and applied to the remaining echoes. Hence, the scaling factor for 4D intensity normalization was similarly computed from the first echo and applied to the remaining echoes. We used a similar preprocessing pipeline for the test data, except the echoes were combined using the above‐mentioned combination Equation (1).

### 2.9 AROMA denoising

AROMA is also an ICA‐based method but does not require a new classifier training for each new data set (Pruim et al., 2015). The pretrained AROMA classifier uses four features from fMRI data sets: high‐frequency content, correlation with realignment parameters, edge fraction, and CSF fraction. Again, each experimental fMRI data set was decomposed into independent components (ICA) and classified as signal or noise using the AROMA classifier. Components identified as noise were consequently regressed out of the original data. The AROMA denoising was performed after fmriprep preprocessing, the noise components were regressed using FSL “REGFILT,” and then the data were filtered and smoothed.

### 2.10 Intraclass correlation coefficients (ICC)

To detect test–retest reliability of activation maps across sessions for different protocols. We calculated intraclass correlation coefficients (ICC) (3.1) as described in (Shrout & Fleiss, 1979). This statistic measured the test–retest reliability of fMRI data (Rombouts et al., 1997, Caceres et al., 2009). A high value (>0.5) indicated that a particular acquisition and analysis approach was highly reproducible across sessions. The ICC values were calculated within the task‐activated regions by masking the contrast maps across subjects. The mask was created utilizing the multi‐subject dictionary learning (MIST) ROI atlas based on resting‐state functional MRI data from 20 subjects with eyes closed (Varoquaux et al., 2011) as shown in Figure S1. The atlas was resampled to 2.5 mm resolution, identical to our data set, using the Nilearn python module (https://nilearn.github.io). The test–retest reliability can determine whether there is a higher variation across different protocols and denoising methods than between subjects. Higher ICC values indicated a better acquisition and analysis strategy. We also examined the *z*‐score distribution across subjects between sessions at first‐level analysis for evaluating the differences in *z*‐scores across protocols (Table 2).

**TABLE 2:** ICC values

Intraclass correlation coefficients (ICC)			
	STANDARD	AROMA	FIX
MB	0.54	0.46	0.53
MBME	0.54	0.51	0.55

*Note*: The table shows ICC values for MB and MBME with different denoising methods. We notice similar ICC values between MB and MBME across different denoising methods; values >0.5 indicate high reproducibility.

Abbreviations: MB factor, slice acceleration factor; MBME, multiband multiecho.

### 2.11 MB and MBME analysis

We conducted a first‐level analysis for each participant with the preprocessed images. For the first‐level analysis, the regressors for the corresponding stimulus were modeled as a boxcar function convolved with the canonical hemodynamic response function. The design matrix contained two regressors modeling the congruent and incongruent conditions against the baseline convolved with a hemodynamic response function and a constant term. We selected each voxel according to SPM's GLM guidelines and parameters (Friston, 2007; Friston et al., 2002). Once the first‐level analysis was completed, we created two contrast images for each explanatory variable (EV), one for congruent conditions [1 0] and one for the incongruent condition [0 1] for each participant for an individual session. Next, we performed the second‐level analysis with classical inference implemented in SPM12. To examine the difference in neural activity for the congruent and incongruent conditions between different protocols and denoising methods. We performed a one‐sample ‐test with contrast images created from the first‐level analysis. All 14 contrast images from each session were entered into a second‐level one‐sample *t*‐test model creating subject‐specific activation maps. Then, we used a classical inference module implemented in SPM12. At the end of the classical inference, we examined which voxels survived thresholds provided by SPM12 by default. The following thresholding criteria were utilized: (1) a cluster‐forming threshold *p* < .001 and a cluster‐wise threshold *p* < .05 [familywise error (FWE) corrected] and (2) a voxel‐wise threshold *p* < .05 (FWE corrected).

The *t*‐maps for each session at first‐level analysis and group‐level were converted to *z*‐maps using FSL (Woolrich et al., 2009). The congruent contrast typically activated visual areas, motor areas from the button press, and some orbitofrontal cortex. The incongruent contrast is essential for identifying deactivation in the frontal medial and default mode network (areas close to the precuneus) (Norris et al., 2002). Furthermore, as the same subjects were scanned twice, we also conducted test–retest reliability on the contrast images created at the first level analysis for each subject for both sessions (see detail below in the Section 4.1).

For comparing protocols across different denoising methods, subject‐specific contrast maps across sessions were used to detect statistically significant differences between different protocols using a paired *t*‐test (*p* < .05, *I* corrected) in SPM12. The *t*‐maps across different protocols and denoising methods were converted to *z*‐maps using FSL (Woolrich et al., 2009) thresholded at (*Z* > 3.1). The purpose of converting *t*‐maps to *z*‐score was to standardize them to compare values of two different distributions.

**FIGURE 2:**
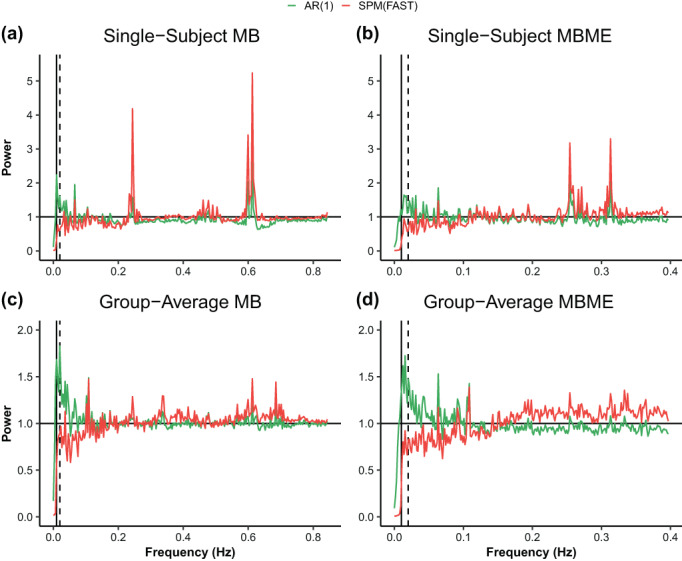
Power spectra of the GLM residuals averaged across brain voxels for standard denoising, AR (1) from FSL shown in green, and SPM (FAST) shown in red. (a) MB for a single‐subject, (b) MBME for single‐subject, (c) MB average across subjects and sessions, (d) MBME average across subjects and sessions. The precise design frequency is shown with a dotted black line across all plots. The cut‐off frequency of 1/100 s is shown with a solid black line. The dotted lines at 0.02 Hz are due to the actual design period being 23 s (23 + 23 s). The frequencies on the *x*‐axis go up to the Nyquist frequency, which is 0.5/repetition time. If, after prewhitening, the residuals were white (as it is assumed), the power spectra would be flat. SPM (FAST) led to the best whitening performance (flatter spectra). For FSL, substantial noise was left after prewhitening, particularly at low frequencies

To account for temporal autocorrelation in the data caused by TR differences between protocols, we opted for “FAST” option in SPM‐12 (Corbin et al., 2018; Friston, 1995). SPM FAST was utilized by correcting for nonsphericity, which is particularly relevant at short TRs, and comparing sequences with different TR. However, before performing the sequence comparison, residuals from the GLM were examined to explore the differences seen between SPM FAST and AR (1) used in FSL in S1. We looked at the power spectrum from single‐subjects and averages across all the subjects and sessions within the residuals. If prewhitening were performed accurately, the power spectrum would be flat. Based on the results shown in Figure 2, substantial noise is left after prewhitening with AR (1) (Bollmann et al., 2018). Hence, we opted to use SPM FAST for analyzing the data.

### 2.12 MB versus MBME comparison

The results from the MB and MBME protocols were examined in three separate different ways:

1. The *z*‐scores at the first level analysis across all subjects for each session were assessed to observe the *z*‐score differences between different protocols across sessions.
2. The *z*‐maps from the group‐level analysis conducting a one‐sample *t*‐test were compared across different denoising methods and protocols to detect any differences in activation: representative examples are labeled with circle as shown in Figure 4.
3. The selected two protocols were quantitatively evaluated using paired *t*‐tests and displaying the statistically significant areas in terms of *z*‐scores.

## 3 RESULTS

**FIGURE 3:**
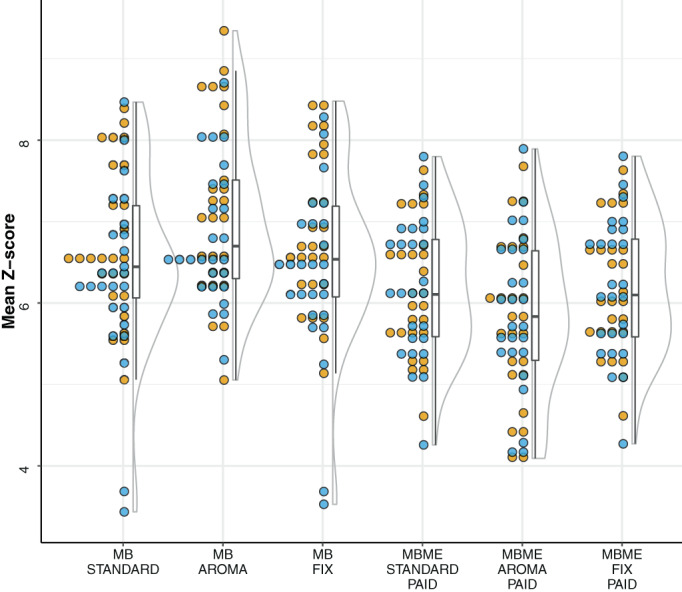
Mean *z*‐scores (>3.1) for all subjects across sessions for different protocols and denoising methods. Color dots represent sessions (yellow and blue stand for Sessions 1 and 2). The distributions on the right show the mean *z*‐scores of methods. Each distribution is based on data from both sessions). The boxplots provide a summary view of the median, 10th, and 90th percentiles of the mean *z*‐score

First, we report our findings obtained on *z*‐scores (>3.1) distribution for all subjects and sessions at first‐level analysis, followed by ICC values for both protocols with and without denoising. Finally, we show results from group‐level analysis of one‐sample and paired *t*‐tests (Figure 3).

**FIGURE 4:**
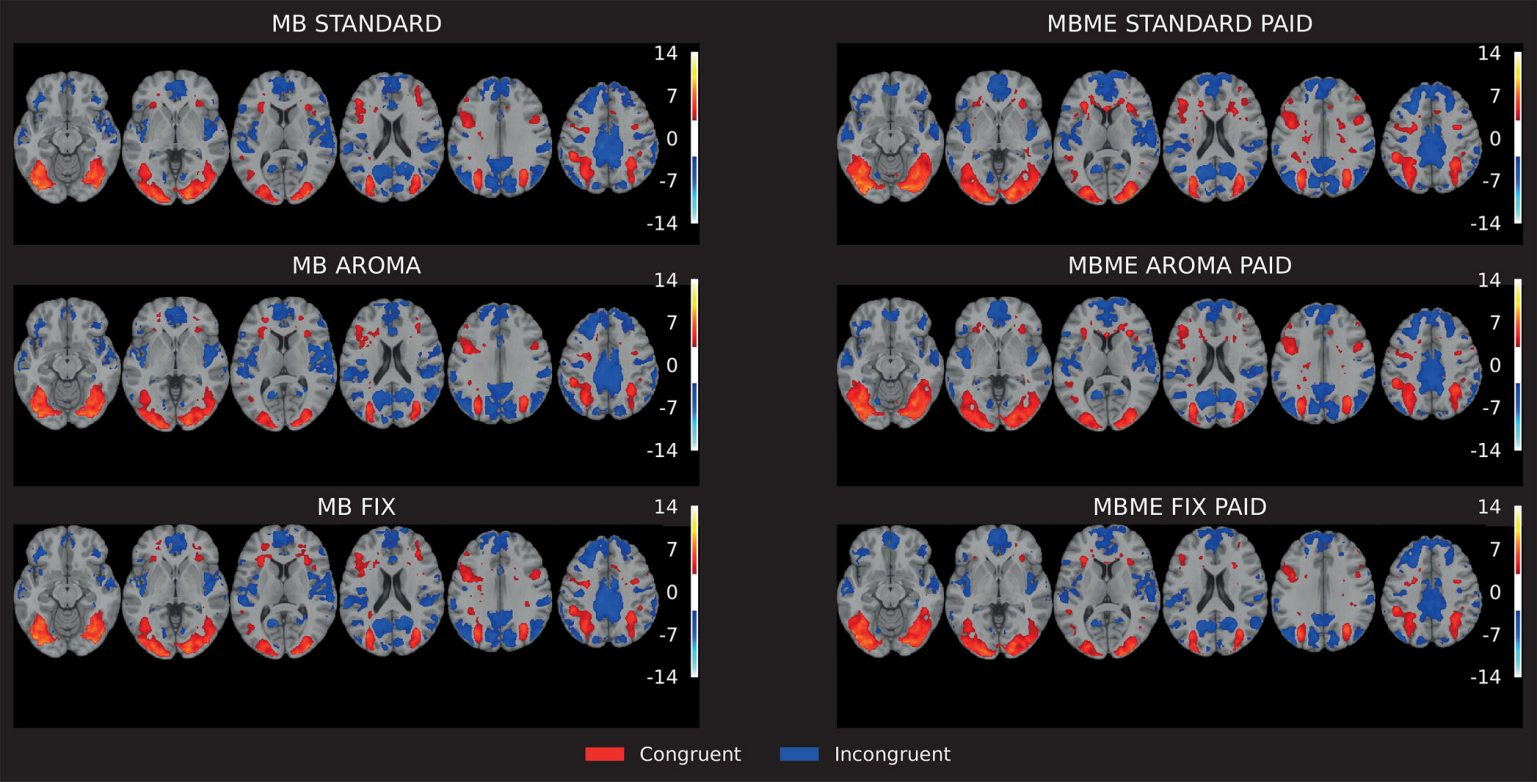
Group activation for MB (left) and MBME PAID (right) using different preprocessing pipelines (top—Standard, middle—AROMA, and bottom—FIX). MBME shows a somewhat higher number of activated voxels in inferior frontal regions than MB, as pointed out by the orange circles compared to cyan circles in MB/ within each protocol, it is apparent that there is no obvious benefit of cleaning. Color range 3.1–8.

Figure 4 shows one‐sample *t*‐test group activation maps for MB and MBME PAID protocols and the two denoising approaches. Although, the activation pattern is similar across protocols in visual, motor, and medial frontal areas, we notice that MBME shows more activation in the inferior medial frontal regions and focal activation in a subcortical region, which seems to be absent in MB. By visual inspection virtually no difference can be seen at the group level for the Standard, AROMA, and FIX denoising strategies in both protocols. We found that the PAID combination shows greater statistical significance across multiple brain regions for MBME (results not shown) than echo summation; therefore, results for MBME are only shown for PAID combination. We found a 50% higher mean *z*‐scores and 82% increase in the number of activated voxels in subcortical regions with MBME Standard PAID compared to MB Standard, 90% higher mean *z*‐scores and 40% more activated voxels within inferior medial prefrontal regions for MBME Standard PAID. We noticed 25% more activated voxels within the precuneus region with similar mean *z*‐scores for MB Standard than MBME Standard PAID (Table 3).

**TABLE 3:** The maximum and minimum range for mean *z*‐scores (*Z* > 3.1) and the number of activated voxels from the group‐level one‐sample *t*‐test averaged across five activated regions selected from MIST parcels for MBME and MB

	STANDARD [min, max]	AROMA [min, max]	FIX [min, max]
Mean *Z*‐scores			
MB	[3.5, 12.3]	[3.5, 12.4]	[3.5, 12.3]
MBME	[6.7, 12.4]	[6.7, 12.1]	[6.8, 11.8]
Number of activated voxels			
MB	[237, 5706]	[222, 5550]	[254, 5881]
MBME	[400, 6289]	[366, 6183]	[173, 5549]

Abbreviations: MB factor, slice acceleration factor; MBME, multiband multiecho.

**FIGURE 5:**
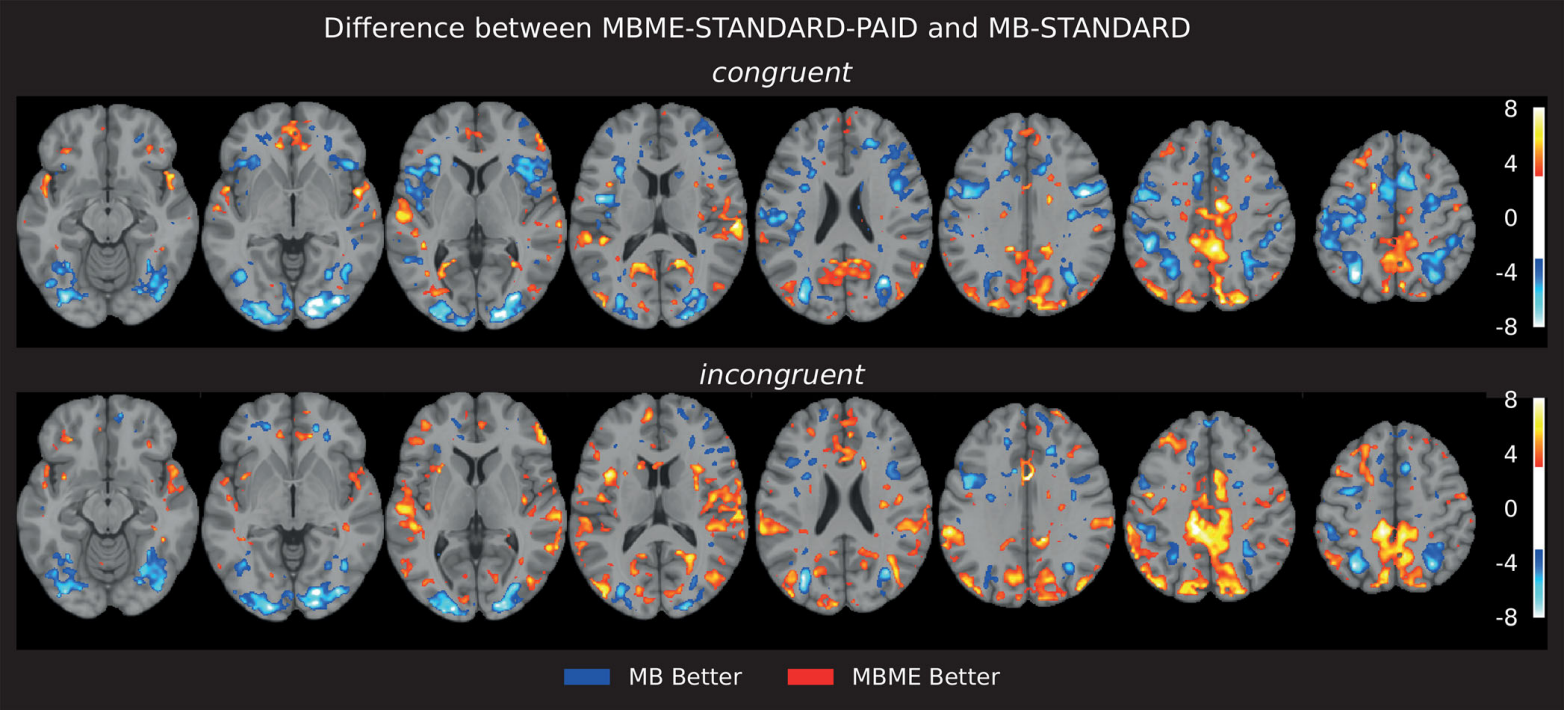
Group activation for MB (left) and MBME PAID (right) using different preprocessing pipelines (top—Standard, middle—AROMA, and bottom—FIX). MBME shows a somewhat higher number of activated voxels in inferior frontal regions than MB, as pointed out by the orange circles compared to cyan circles in MB/within each protocol, it is apparent that there is no obvious benefit of cleaning. Color range 3.1–8.

Figure 5 shows results from the sequence comparison using paired *t*‐test (FDR, *p* < .05) between MB‐STANDARD and MBME‐STANDARD‐PAID (with standard denoising) for each of the two contrasts considered (congruent, incongruent). In the medial prefrontal cortex and cortical areas, that is, regions related to the “Default Mode Network,” MBME performs better than MB, in terms of significantly higher *z*‐scores. There is also a small visible cluster of activation seen in the subcortical areas for MBME only. MB performs better within the visual and anterior cingulate cortex The trend observed is independent of the denoising method, that is, AROMA and STANDARD (results not shown).

## 4 DISCUSSION

In the current study, we compared MB and MBME in terms of their sensitivity for task fMRI. The data sets from both protocols were reconstructed and preprocessed with identical pipelines. The data from 14 healthy subjects were acquired in two sessions, using an adapted color‐word matching Stroop task (Boyacioğlu et al., 2014), which induces widespread activation throughout the brain. The performance was assessed by (i) evaluating *z*‐scores across sessions to observe differences between protocols and denoising methods. (ii) By examining *z*‐scores within the activated regions at the group level analysis and (iii) by looking into the difference between the parameter estimates using paired *t*‐tests across different protocols. We used a minimal denoising strategy (“Standard”) and two widely used ICA‐based denoising strategies, ICA‐AROMA and ICA‐FIX.

The results indicated high test–retest reliability within each protocol, as seen by the *z*‐score distribution across sessions and ICC values (see Table 2). At the group‐level one‐sample *t*‐test, statistics were computed to examine how the spatial distribution and extent of the detected activation compared between the two sequences. Within frontal and subcortical areas, MBME showed a greater extent of activation. MBME Standard PAID came out better than MBME with FIX and AROMA, MB generally showed comparable performance between different denoising methods. A paired *t*‐test was performed between MBME and MB to compare both protocols at the group level, which confirmed that MBME performed better within subcortical, prefrontal and motor areas compared to MB. However, MB performed better within visual occipital areas compared to MBME (see Figure 5).

Our results indicate that MBME acquisition has a higher sensitivity in the areas affected by dropout and inhomogeneity. As demonstrated previously, multiecho fMRI is somewhat more sensitive than standard EPI (Poser et al., 2006). We also noticed no significant sensitivity gain for ICA‐FIX and ICA‐AROMA than the Standard denoising pipeline for MBME (see Figure 4).One possible explanation is that denoising approaches tend to decrease reliability by removing the common noise sources that are highly reliable and increasing the validity of results (Noble et al., 2019). The present contribution shows that acquiring data at sub‐second TR gives no significant improvement in statistical *z*‐scores as *z*‐scores values between MB and MBME were comparable at first‐level analysis (see Figure 3).

### 4.1 Intraclass correlation coefficients

We observed both protocols to be moderately reproducible across sessions. The ICC for MB‐AROMA is the lowest. ICA‐AROMA removes motion‐related artifacts that can vary across sessions, and MB‐EPI may exhibit increased motion sensitivity due to short TR. As seen in Figure 3, we observed a slight variation in *z*‐scores across sessions, suggesting low intrasubject variability within the protocol.

### 4.2 Denoising approaches

The fundamental reason behind the widespread adoption of “data‐driven” approaches over physiological monitoring data is, if available or collected, the latter are not expected to relate to all common forms of artifact (e.g., scanner artifacts). The HCP used FIX (Griffanti et al., 2014) to denoise resting‐state fMRI data acquired with MB and found improved *z*‐scores across resting‐state networks, which led to the broader application of ICA‐FIX in fMRI. However, in task‐fMRI, we found no significant gains of the FIX classifier for both protocols. A possible explanation is applying a proper AR model order, which removes autocorrelated structure from the data, most of which is related to physiological noise and motion. The physiological noise removal reduces the required AR model order, but the remaining serial correlations need an advanced model. Furthermore, ICA‐FIX can reduce sensitivity (data not shown) due to the loss of a higher number of degrees of freedom compared to other denoising methods. It focuses on removing motion and physiological noise. There is also a considerable overhead in using FIX because of the requirement for acquiring and training data for at least 10 subjects (https://fsl.fmrib.ox.ac.uk/fsl/fslwiki/FIX). In contrast, ICA‐AROMA required no additional training and retained the signal of interest, demonstrating greater generalizability.

### 4.3 PAID (CNR) weighting in MBME

The results from two different echo combination schemes showed PAID weighting performed better than simple summation. This finding is consistent with that of (Poser et al., 2006), where PAID weighing increased CNR. The PAID weighting showed a 10% higher tSNR for MBME as compared to simple summation. Furthermore, it improved the group analysis by increasing the sensitivity and detection of small activation clusters within the subcortical areas, and therefore, should be preferred to simple averaging. This result contrasts with the null results previously reported with a double‐echo protocol (Kettinger et al., 2016).

### 4.4 Temporal autocorrelation

With readily available acquisition protocols and online reconstruction for MB sequences, there is a tendency toward using MB fMRI as the default acquisition protocol. One aspect often neglected is the appropriate correction of temporal autocorrelation. A higher sampling rate leads to a higher temporal autocorrelation in the data, in which the effective degrees of freedom is smaller than the number of samples acquired. If left uncorrected, this can lead to inflated *z*‐scores (Todd et al., 2017), increased false positives (Olszowy et al., 2019), and an overestimation of power (Mumford, 2012). In FSL, task fMRI data is usually prewhitened using an AR (1)‐model, which is considered sufficient for short TR's. However, for TRs, less than a second, a higher rate of false positives has previously been observed (Olszowy et al., 2019). A limited number of studies (Bollmann et al., 2018; Olszowy et al., 2019; Todd et al., 2016) investigated using an appropriate AR model for task fMRI currently implemented in SPM as the “FAST” option for short TR data. In the current study, we corrected the temporal autocorrelation by incorporating the actual degrees of freedom in the *t*‐scores for MB and MBME using SPM “FAST” and found the difference between short TR *z*‐scores seems to be minimal between protocols.

### 4.5 Protocol choices

The analysis techniques and the brain regions of interest in task fMRI can influence the choice of data acquisition protocol. There is a considerable gain from a higher MB factor for methods, such as multivariate pattern analysis. The higher number of data points from MB produces robust (i.e., less noisy) estimates, leading to a more reliable classifier performance than the ROI‐ or GLM‐based approach (Demetriou et al., 2018). However, for GLM‐based approaches, MBME has previously been shown to have higher sensitivity in regions with dropouts and distortions (Boyacioğlu et al., 2015; Kirilina et al., 2016; Poser et al., 2006). This study is consistent with previous research (Kirilina et al., 2016), which compared standard and ME‐EPI's sensitivity using emotional and reward‐based learning tasks. ME‐EPI performed better in regions where the single‐echo protocol suffered dropouts, namely, in an ROI placed at the orbitofrontal cortex. A similar conclusion was reached by Puckett et al. (2018), where MBME performed better than a single‐echo within iron‐rich areas in the subcortical regions. A recent comparison of MBME with MB using a visual checkerboard showed high activation volume and high sensitivity for MBME (Cohen et al., 2021). We can infer that MBME performs better than MB in the areas affected by dropout and susceptibility induced inhomogeneity. One potential limitation for MBME‐EPI is the longer ETL: this limits both the voxel size and achievable TR as well as necessitating in‐plane parallel imaging and partial Fourier acquisition. In this study, both sequences were optimized to acquire signals as efficiently as possible, and reconstruction artifacts did not compromise the signal quality.

## 5 CONCLUSION

In summary, with this comparison study, we found that MBME and MB give comparable performance in most brain regions. In the regions with dropouts and susceptibility induced inhomogeneity MBME performed somewhat better at the group level. The results showed that the difference between sequences is more significant than the difference between different denoising (FIX, AROMA) strategies at the group level, highlighting the importance of considering an optimized sequence before data collection. For MBME: the choice of an optimal echo combination can influence the sensitivity at the group‐level analysis.

## Supporting information


**Appendix S1** Supplementary Information

## Data Availability

Our data and analysis pipelines will be publicly available upon acceptance in our Institute's Data Repository. The Donders Repository is the repository for sharing data of Donders Institute researchers. The researcher can share the storage with the author/journal upon request. For which, the data sharing should hold the following principles. (1) Data sets that potentially contain personally identifiable information can only be shared under a Data Use Agreement that protects the identity of the participants (e.g., Data Use Agreement forbids to establish identity or to attempt to contact participants) and (2) the rights holder remains as defined earlier in the project. To share the research data with researchers other than collaborators, we can ask for Data Sharing Collection (DSC). By publishing a DSC, the researcher's data are made publicly available.

## References

[hbm26081-bib-0079] Bandettini, P. A., Wong, E. C., Hinks, R. S., Tikofsky, R. S., & Hyde, J. S. (1992). Time course EPI of human brain function during task activation. Magnetic Resonance in Medicine, 25(2), 390–397. 10.1002/mrm.1910250220 1614324

[hbm26081-bib-0002] Bollmann, S. , Puckett, A. M. , Cunnington, R. , & Barth, M. (2018). Serial correlations in single‐subject fMRI with sub‐second TR. NeuroImage, 166(February), 152–166. 10.1016/J.NEUROIMAGE.2017.10.043 29066396

[hbm26081-bib-0003] Boyacioğlu, R. , Schulz, J. , Koopmans, P. J. , Barth, M. , & Norris, D. G. (2015). Improved sensitivity and specificity for resting state and task fMRI with multi‐band multi‐echo EPI compared to multi‐echo EPI at 7T. NeuroImage, 119, 352–361. 10.1016/j.neuroimage.2015.06.089 26162554

[hbm26081-bib-0004] Boyacioğlu, R. , Schulz, J. , Müller, N. C. J. , Koopmans, P. J. , Barth, M. , & Norris, D. G. (2014). Whole brain, high resolution multi‐band spin‐echo EPI fMRI at 7 T: a comparison with gradient‐echo EPI using a color‐word Stroop task. NeuroImage, 97, 142–150. 10.1016/j.neuroimage.2014.04.011 24736172

[hbm26081-bib-0005] Breuer, F. A. , Blaimer, M. , Mueller, M. F. , Seiberlich, N. , Heidemann, R. M. , Griswold, M. A. , & Jakob, P. M. (2006). Controlled aliasing in volumetric parallel imaging (2D CAIPIRINHA). Magnetic Resonance in Medicine, 55, 549–556. 10.1002/mrm.20787 16408271

[hbm26081-bib-0082] Caceres, A., Hall, D. L., Zelaya, F. O., Williams, S. C. R., & Mehta, M. A. (2009). Measuring fMRI reliability with the intra‐class correlation coefficient. NeuroImage, 45(3), 758–768. 10.1016/j.neuroimage.2008.12.035 19166942

[hbm26081-bib-0006] Cauley, S. F. , Polimeni, J. R. , Bhat, H. , Wald, L. L. , & Setsompop, K. (2014). Interslice leakage artifact reduction technique for simultaneous multislice acquisitions. Magnetic Resonance in Medicine, 72(1), 93–102. 10.1002/mrm.24898 23963964 PMC4364522

[hbm26081-bib-0007] Chen, L. , Vu, A. T. , Xu, J. , Moeller, S. , Ugurbil, K. , Yacoub, E. , & Feinberg, D. A. (2015). Evaluation of highly accelerated simultaneous multislice EPI for fMRI. NeuroImage, 104, 452–459. 10.1016/j.neuroimage.2014.10.027 25462696 PMC4467797

[hbm26081-bib-0009] Cohen, A. D. , Jagra, A. S. , Yang, B. , Fernandez, B. , Banerjee, S. , & Wang, Y. (2021). Detecting task functional MRI activation using the multiband multiecho (MBME) echo‐planar imaging (EPI) sequence. Journal of Magnetic Resonance Imaging, 53, 1366–1374. 10.1002/JMRI.27448 33210793 PMC10937038

[hbm26081-bib-0010] Corbin, N. , Todd, N. , Friston, K. J. , & Callaghan, M. F. (2018). Accurate modeling of temporal correlations in rapidly sampled fMRI time series. Human Brain Mapping, 39, 3884–3897. 10.1002/hbm.24218 29885101 PMC6175228

[hbm26081-bib-0011] de Zwart, J. A. , van Gelderen, P. , Kellman, P. , & Duyn, J. H. (2002). Application of sensitivity‐encoded echo‐planar imaging for blood oxygen level‐dependent functional brain imaging. Magnetic Resonance in Medicine, 48(6), 1011–1020. 10.1002/mrm.10303 12465111

[hbm26081-bib-0012] Demetriou, L. , Kowalczyk, O. S. , Tyson, G. , Bello, T. , Newbould, R. D. , & Wall, M. B. (2018). A comprehensive evaluation of increasing temporal resolution with multiband‐accelerated protocols and effects on statistical outcome measures in fMRI. NeuroImage, 176, 404–416. 10.1016/J.NEUROIMAGE.2018.05.011 29738911

[hbm26081-bib-0013] Dipasquale, O. , Sethi, A. , Laganà, M. M. , Baglio, F. , Baselli, G. , Kundu, P. , Harrison, N. A. , & Cercignani, M. (2017). Comparing resting state fMRI de‐noising approaches using multi‐ and single‐echo acquisitions. PLoS One, 12, e0173289. 10.1371/journal.pone.0173289 28323821 PMC5360253

[hbm26081-bib-0014] Esteban, O. , Blair, R. , Markiewicz, C. J. , Berleant, S. L. , Moodie, C. , Ma, F. , Isik, A. I. , Erramuzpe, A. , Kent, J. D. , Goncalves, M. , DuPre, E. , Sitek, K. R. , Poldrack, R. A. , & Gorgolewski, K. J. (2018). Poldracklab/Fmriprep: 1.0.8. 10.5281/ZENODO.1183390

[hbm26081-bib-0015] Feinberg, D. A. , Moeller, S. , Smith, S. M. , Auerbach, E. , Ramanna, S. , Glasser, M. F. , Miller, K. L. , Ugurbil, K. , & Yacoub, E. (2010). Multiplexed echo planar imaging for sub‐second whole brain fmri and fast diffusion imaging. PLoS One, 5, e15710. 10.1371/journal.pone.0015710 21187930 PMC3004955

[hbm26081-bib-0017] Friston, K. (1995). Analysis of fMRI time‐series revisited. NeuroImage, 2, 45–53. 10.1006/nimg.1995.1007 9343589

[hbm26081-bib-0018] Friston, K. (2007). Statistical parametric mapping. In K. J. Friston , J. Ashburner , S. Kiebel , T. Nichols , & W. D. Penny (Eds.), Statistical parametric mapping: The analysis of funtional brain images (pp. 10–31). Elsevier/Academic Press.

[hbm26081-bib-0019] Friston, K. J. , Glaser, D. E. , Henson, R. N. A. , Kiebel, S. , Phillips, C. , & Ashburner, J. (2002). Classical and Bayesian inference in neuroimaging: Applications. NeuroImage, 16, 484–512. 10.1006/nimg.2002.1091 12030833

[hbm26081-bib-0020] Friston, K. J. , Holmes, A. P. , Worsley, K. J. , Poline, J.‐P. , Frith, C. D. , & Frackowiak, R. S. J. (1994). Statistical parametric maps in functional imaging: A general linear approach. Human Brain Mapping, 2(4), 189–210. 10.1002/hbm.460020402

[hbm26081-bib-0021] Gomez, E. D. , Boyacioğlu, R. , Schulz, J. , Marques, J. P. , Norris, D. G. , & Poser, B. A. (2015). Multi‐echo multi‐band EPI: Preliminary results from a protocol comparison rsfMRI study. In Proceedings of the ESMRMB. 6–7.

[hbm26081-bib-0022] Gorgolewski, K. , Burns, C. D. , Madison, C. , Clark, D. , Halchenko, Y. O. , Waskom, M. L. , & Ghosh, S. S. (2011). Nipype: A flexible, lightweight and extensible neuroimaging data processing framework in python. Frontiers in Neuroinformatics, 5, 13. 10.3389/fninf.2011.00013 21897815 PMC3159964

[hbm26081-bib-0023] Gorgolewski, K. J. , Auer, T. , Calhoun, V. D. , Craddock, R. C. , Das, S. , Duff, E. P. , Flandin, G. , Ghosh, S. S. , Glatard, T. , Halchenko, Y. O. , Handwerker, D. A. , Hanke, M. , Keator, D. , Li, X. , Michael, Z. , Maumet, C. , Nichols, B. N. , Nichols, T. E. , Pellman, J. , … Poldrack, R. A. (2016). The brain imaging data structure, a format for organizing and describing outputs of neuroimaging experiments. Scientific Data, 3, 160044. 10.1038/sdata.2016.44 27326542 PMC4978148

[hbm26081-bib-0024] Greve, D. N. , & Fischl, B. (2009). Accurate and robust brain image alignment using boundary‐based registration. NeuroImage, 48(1), 63–72. 10.1016/J.NEUROIMAGE.2009.06.060 19573611 PMC2733527

[hbm26081-bib-0025] Griffanti, L. , Douaud, G. , Bijsterbosch, J. , Evangelisti, S. , Alfaro‐Almagro, F. , Glasser, M. F. , Duff, E. P. , Fitzgibbon, S. , Westphal, R. , Carone, D. , Beckmann, C. F. , & Smith, S. M. (2017). Hand classification of fMRI ICA noise components. NeuroImage, 154, 188–205. 10.1016/j.neuroimage.2016.12.036 27989777 PMC5489418

[hbm26081-bib-0026] Griffanti, L. , Salimi‐Khorshidi, G. , Beckmann, C. F. , Auerbach, E. J. , Douaud, G. , Sexton, C. E. , Zsoldos, E. , Ebmeier, K. P. , Filippini, N. , Mackay, C. E. , Moeller, S. , Xu, J. , Yacoub, E. , Baselli, G. , Ugurbil, K. , Miller, K. L. , & Smith, S. M. (2014). ICA‐based artefact removal and accelerated fMRI acquisition for improved resting state network imaging. NeuroImage, 95(July), 232–247. 10.1016/J.NEUROIMAGE.2014.03.034 24657355 PMC4154346

[hbm26081-bib-0027] Griffeth, V. E. M. , Blockley, N. P. , Simon, A. B. , & Buxton, R. B. (2013). A new functional MRI approach for investigating modulations of brain oxygen metabolism. PLoS One, 8(6), e68122. 10.1371/journal.pone.0068122 23826367 PMC3694916

[hbm26081-bib-0028] Griswold, M. A. , Jakob, P. M. , Heidemann, R. M. , Nittka, M. , Jellus, V. , Wang, J. , Kiefer, B. , & Haase, A. (2002). Generalized autocalibrating partially parallel acquisitions (GRAPPA). Magnetic Resonance in Medicine, 47(6), 1202–1210. 10.1002/mrm.10171 12111967

[hbm26081-bib-0029] Hagberg, G. E. , Indovina, I. , Sanes, J. N. , & Posse, S. (2002). Real‐time quantification ofT2* changes using multiecho planar imaging and numerical methods. Magnetic Resonance in Medicine, 48(5), 877–882. 10.1002/mrm.10283 12418003

[hbm26081-bib-0030] Huntenburg, J. M . (2014). Evaluating nonlinear coregistration of BOLD EPI and T1w images. https://pure.mpg.de/pubman/faces/ViewItemOverviewPage.jsp?itemId=item{\_}2327525

[hbm26081-bib-0031] Jenkinson, M. , Bannister, P. , Brady, M. , & Smith, S. (2002). Improved optimization for the robust and accurate linear registration and motion correction of brain images. NeuroImage, 17(2), 825–841. 10.1006/NIMG.2002.1132 12377157

[hbm26081-bib-0032] Jenkinson, M. , & Smith, S. (2001). A global optimisation method for robust affine registration of brain images. Medical Image Analysis, 5(2), 143–156. 10.1016/s1361-8415(01)00036-6 11516708

[hbm26081-bib-0033] Kettinger, Á. , Hill, C. , Vidnyánszky, Z. , Windischberger, C. , & Nagy, Z. (2016). Investigating the group‐level impact of advanced dual‐echo fMRI combinations. Frontiers in Neuroscience, 10, 571. 10.3389/fnins.2016.00571 28018165 PMC5149566

[hbm26081-bib-0034] Kirilina, E. , Lutti, A. , Poser, B. A. , Blankenburg, F. , & Weiskopf, N. (2016). The quest for the best: The impact of different EPI sequences on the sensitivity of random effect fMRI group analyses. NeuroImage, 126, 49–59. 10.1016/J.NEUROIMAGE.2015.10.071 26515905 PMC4739510

[hbm26081-bib-0035] Kwong, K. K. , Belliveau, J. W. , Chesler, D. A. , Goldberg, I. E. , Weisskoff, R. M. , Poncelet, B. P. , Kennedy, D. N. , Hoppel, B. E. , Cohen, M. S. , & Turner, R. (1992). Dynamic magnetic resonance imaging of human brain activity during primary sensory stimulation. Proceedings of the National Academy of Sciences of the United States of America, 89(12), 5675–5679. 10.1073/pnas.89.12.5675 1608978 PMC49355

[hbm26081-bib-0036] Larkman, D. J. , Hajnal, J. V. , Herlihy, A. H. , Coutts, G. A. , Young, I. R. , & Ehnholm, G. (2001). Use of multicoil arrays for separation of signal from multiple slices simultaneously excited. Journal of Magnetic Resonance Imaging, 13(2), 313–317. 10.1002/1522-2586(200102)13:2<313::AID-JMRI1045>3.0.CO;2-W 11169840

[hbm26081-bib-0038] Li, X. , Morgan, P. S. , Ashburner, J. , Smith, J. , & Rorden, C. (2016). The first step for neuroimaging data analysis: DICOM to NIfTI conversion. Journal of Neuroscience Methods, 264, 47–56. 10.1016/j.jneumeth.2016.03.001 26945974

[hbm26081-bib-0040] Mansfield, P. (1977). Multi‐planar image formation using NMR spin echoes. Journal of Physics C: Solid State Physics, 10, L55.

[hbm26081-bib-0042] Menon, R. S. , Ogawa, S. , Tank, D. W. , & Uğurbil, K. (1993). Tesla gradient recalled echo characteristics of photic stimulation‐induced signal changes in the human primary visual cortex. Magnetic Resonance in Medicine, 30(3), 380–386. 10.1002/mrm.1910300317 8412612

[hbm26081-bib-0043] Moeller, S. , Yacoub, E. , Olman, C. A. , Auerbach, E. , Strupp, J. , Harel, N. , & Uǧurbil, K. (2010). Multi‐band multislice GE‐EPI at 7 tesla, with 16‐fold acceleration using partial parallel imaging with application to high spatial and temporal whole‐brain FMRI. Magnetic Resonance in Medicine, 63(5), 1144–1153. 10.1002/mrm.22361 20432285 PMC2906244

[hbm26081-bib-0044] Mumford, J. A. (2012). A power calculation guide for fMRI studies. Social Cognitive and Affective Neuroscience, 7(6), 738–742. 10.1093/scan/nss059 22641837 PMC3427872

[hbm26081-bib-0045] Noble, S. , Scheinost, D. , & Todd Constable, R. (2019). A decade of test‐retest reliability of functional connectivity: a systematic review and meta‐analysis HHS public access. NeuroImage, 203, 116157. 10.1016/j.neuroimage.2019.116157 31494250 PMC6907736

[hbm26081-bib-0046] Norris, D. G. , Zysset, S. , Mildner, T. , & Wiggins, C. J. (2002). An investigation of the value of spin‐echo‐based fMRI using a Stroop color‐word matching task and EPI at 3 T. NeuroImage, 15(3), 719–726. 10.1006/NIMG.2001.1005 11848715

[hbm26081-bib-0047] Nunes, R. G. , Hajnal, J. V. , Golay, X. , & Larkman, D. J. (2006). Simultaneous slice excitation and reconstruction for single shot EPI. Proceedings of the International Society for Magnetic Resonance in Medicine, 14(2), 293.

[hbm26081-bib-0048] Ogawa, S. , Lee, T. M. , Kay, A. R. , & Tank, D. W. (1990). Brain magnetic resonance imaging with contrast dependent on blood oxygenation. Proceedings of the National Academy of Sciences of the United States of America, 87(24), 9868–9872. 10.1073/pnas.87.24.9868 2124706 PMC55275

[hbm26081-bib-0049] Olafsson, V. , Kundu, P. , Wong, E. C. , Bandettini, P. A. , & Liu, T. T. (2015). Enhanced identification of BOLD‐like components with multi‐echo simultaneous multislice (MESMS) fMRI and multi‐echo ICA. NeuroImage, 112, 43–51. 10.1016/j.neuroimage.2015.02.052 25743045 PMC4408238

[hbm26081-bib-0050] Olszowy, W. , Aston, J. , Rua, C. , & Williams, G. B. (2019). Accurate autocorrelation modeling substantially improves fMRI reliability. Nature Communications, 10(1), 1220. 10.1038/s41467-019-09230-w PMC6428826 30899012

[hbm26081-bib-0051] Polimeni, J. R. , Bhat, H. , Witzel, T. , Benner, T. , Feiweier, T. , Inati, S. J. , Renvall, V. , Heberlein, K. , & Wald, L. L. (2016). Reducing sensitivity losses due to respiration and motion in accelerated echo planar imaging by reordering the autocalibration data acquisition. Magnetic Resonance in Medicine, 75, 665–679. 10.1002/mrm.25628 25809559 PMC4580494

[hbm26081-bib-0052] Poser, B. A. , & Norris, D. G. (2009). Investigating the benefits of multi‐echo EPI for fMRI at 7 T. NeuroImage, 45, 1162–1172. 10.1016/j.neuroimage.2009.01.007 19349231

[hbm26081-bib-0053] Poser, B. A. , Versluis, M. J. , Hoogduin, J. M. , & Norris, D. G. (2006). BOLD contrast sensitivity enhancement and artifact reduction with multiecho EPI: Parallel‐acquired inhomogeneity‐desensitized fMRI. Magnetic Resonance in Medicine, 55(6), 1227–1235. 10.1002/mrm.20900 16680688

[hbm26081-bib-0054] Posse, S. , Wiese, S. , Gembris, D. , Mathiak, K. , Kessler, C. , Grosse‐Ruyken, M. L. , Elghahwagi, B. , Richards, T. , Dager, S. R. , & Kiselev, V. G. (1999). Enhancement of BOLD‐contrast sensitivity by single‐shot multi‐echo functional MR imaging. Magnetic Resonance in Medicine, 42(1), 87–97. 10.1002/(sici)1522-2594(199907)42:1<87::aid-mrm13>3.0.co;2-o 10398954

[hbm26081-bib-0056] Pruessmann, K. P. , Weiger, M. , Scheidegger, M. B. , & Boesiger, P. (1999). SENSE: Sensitivity encoding for fast MRI. Magnetic Resonance in Medicine, 42(5), 952–962. 10542355

[hbm26081-bib-0057] Pruim, R. H. R. , Mennes, M. , Buitelaar, J. K. , & Beckmann, C. F. (2014). Evaluation of ICA‐AROMA and alternative strategies for motion artifact removal in resting‐state fMRI. NeuroImage, 112, 278–287. 10.1016/j.neuroimage.2015.02.063 25770990

[hbm26081-bib-0058] Pruim, R. H. R. , Mennes, M. , van Rooij, D. , Llera, A. , Buitelaar, J. K. , & Beckmann, C. F. (2015). ICA‐AROMA: A robust ICA‐based strategy for removing motion artifacts from fMRI data. NeuroImage, 112, 267–277. 10.1016/J.NEUROIMAGE.2015.02.064 25770991

[hbm26081-bib-0059] Puckett, A. M. , Bollmann, S. , Poser, B. A. , Palmer, J. , Barth, M. , & Cunnington, R. (2018). Using multi‐echo simultaneous multislice (SMS) EPI to improve functional MRI of the subcortical nuclei of the basal ganglia at ultra‐high field (7T). NeuroImage, 172, 886–895. 10.1016/J.NEUROIMAGE.2017.12.005 29208571

[hbm26081-bib-0060] Rombouts, S. , Barkhof, F. , Hoogenraad, F. G. , Sprenger, M. , Valk, J. , & Scheltens, P. (1997). Test‐retest analysis with functional MR of the activated area in the human visual cortex. American Society of Neuroradiology, 18, 1317–1322. PMC8338037 9282862

[hbm26081-bib-0061] Saban, W. , Gabay, S. , & Kalanthroff, E. (2018). More than just channeling: The role of subcortical mechanisms in executive functions—Evidence from the Stroop task. Acta Psychologica, 189, 36–42. 10.1016/J.ACTPSY.2017.03.001 28291524

[hbm26081-bib-0062] Salimi‐Khorshidi, G. , Douaud, G. , Beckmann, C. F. , Glasser, M. F. , Griffanti, L. , & Smith, S. M. (2014). Automatic denoising of functional MRI data: Combining independent component analysis and hierarchical fusion of classifiers. NeuroImage, 90, 449–468. 10.1016/j.neuroimage.2013.11.046 24389422 PMC4019210

[hbm26081-bib-0063] Setsompop, K. , Gagoski, B. A. , Polimeni, J. R. , Witzel, T. , Wedeen, V. J. , & Wald, L. L. (2012). Blipped‐controlled aliasing in parallel imaging for simultaneous multislice echo planar imaging with reduced g‐factor penalty. Magnetic Resonance in Medicine, 67(5), 1210–1224. 10.1002/mrm.23097 21858868 PMC3323676

[hbm26081-bib-0081] Shrout, P. E., & Fleiss, J. L. (1979). Intraclass correlations: Uses in assessing rater reliability. Psychological Bulletin, 86(2), 420–428. 10.1037/0033-2909.86.2.420 18839484

[hbm26081-bib-0064] Smith, S. M. , & Brady, J. M. (1997). SUSAN—A new approach to low level image processing. International Journal of Computer Vision, 23(1), 45–78. 10.1023/A:1007963824710

[hbm26081-bib-0065] Sodickson, D. K. , & Manning, W. J. (1997). Simultaneous acquisition of spatial harmonics (SMASH): Fast imaging with radiofrequency coil arrays. Magnetic Resonance in Medicine, 38(4), 591–603. 10.1002/mrm.1910380414 9324327

[hbm26081-bib-0066] Speck, O. , & Hennig, J. (1998). Functional imaging by *I* _0_‐ and *T* _2_*‐parameter mapping using multi‐image EPI. Magnetic Resonance in Medicine, 40(2), 243–248. 10.1002/mrm.1910400210 9702706

[hbm26081-bib-0067] Todd, N. , Josephs, O. , Zeidman, P. , Flandin, G. , Moeller, S. , & Weiskopf, N. (2017). Functional sensitivity of 2D simultaneous multi‐slice echo‐planar imaging: Effects of acceleration on g‐factor and physiological noise. Frontiers in Neuroscience, 11, 158. 10.3389/fnins.2017.00158 28424572 PMC5372803

[hbm26081-bib-0068] Todd, N. , Moeller, S. , Auerbach, E. J. , Yacoub, E. , Flandin, G. , & Weiskopf, N. (2016). Evaluation of 2D multi‐band EPI imaging for high‐resolution, whole‐brain, task‐based fMRI studies at 3T: Sensitivity and slice leakage artifacts. NeuroImage, 124, 32–42. 10.1016/j.neuroimage.2015.08.056 26341029 PMC4655914

[hbm26081-bib-0069] Treiber, J. M. , White, N. S. , Steed, T. C. , Bartsch, H. , Holland, D. , Farid, N. , McDonald, C. R. , Carter, B. S. , Dale, A. M. , & Chen, C. C. (2016). Characterization and correction of geometric distortions in 814 diffusion weighted images. PLoS One, 11(3), e0152472. 10.1371/journal.pone.0152472 27027775 PMC4814112

[hbm26081-bib-0080] Triantafyllou, C., Hoge, R. D., Krueger, G., Wiggins, C. J., Potthast, A., Wiggins, G. C., & Wald, L. L. (2005). Comparison of physiological noise at 1.5 T, 3 T and 7 T and optimization of fMRI acquisition parameters. NeuroImage, 26(1), 243–250. 10.1016/j.neuroimage.2005.01.007 15862224

[hbm26081-bib-0071] Uğurbil, K. , Xu, J. , Auerbach, E. J. , Moeller, S. , Vu, A. T. , Duarte‐Carvajalino, J. M. , Lenglet, C. , Wu, X. , Schmitter, S. , Van de Moortele, P. F. , Strupp, J. , Sapiro, G. , De Martino, F. , Wang, D. , Harel, N. , Garwood, M. , Chen, L. , Feinberg, D. A. , Smith, S. M. , … WU‐Minn HCP Consortium . (2013). Pushing spatial and temporal resolution for functional and diffusion MRI in the human connectome project. NeuroImage, 80, 80–104. 10.1016/j.neuroimage.2013.05.012 23702417 PMC3740184

[hbm26081-bib-0083] Varoquaux, G., Gramfort, A., Pedregosa, F., Michel, V., & Thirion, B. (2011). Multi‐subject Dictionary Learning to Segment an Atlas of Brain Spontaneous Activity. Information Processing in Medical Imaging, 562–573. 10.1007/978-3-642-22092-0_46 21761686

[hbm26081-bib-0074] Wang, S. , Peterson, D. J. , Gatenby, J. C. , Li, W. , Grabowski, T. J. , & Madhyastha, T. M. (2017). Evaluation of field map and nonlinear registration methods for correction of susceptibility artifacts in diffusion MRI. Frontiers in Neuroinformatics, 11, 17. 10.3389/fninf.2017.00017 28270762 PMC5318394

[hbm26081-bib-0076] Woolrich, M. W. , Jbabdi, S. , Patenaude, B. , Chappell, M. , Makni, S. , Behrens, T. , Beckmann, C. , Jenkinson, M. , & Smith, S. M. (2009). Bayesian analysis of neuroimaging data in FSL. NeuroImage, 45(1), S173–S186. 10.1016/j.neuroimage.2008.10.055 19059349

[hbm26081-bib-0078] Zysset, S. , Mü, K. , Lohmann, G. , & von Cramon, D. Y. (2001). Color‐word matching Stroop task: Separating interference and response conflict. NeuroImage, 13(1), 29–36. 10.1006/nimg.2000.0665 11133306

