# Supplementary material for: A comparison of multiband and multiband multiecho gradient‐echo EPI for task fMRI at 3 T
Source: Hum Brain Mapp. 2022 Oct 5;44(1):82–93. doi: 10.1002/hbm.26081 (PMC9783458; doi:10.1002/hbm.26081)
Supplement: Supplementary file 1 — Appendix S1 Supplementary Information [file HBM-44-82-s001.docx]

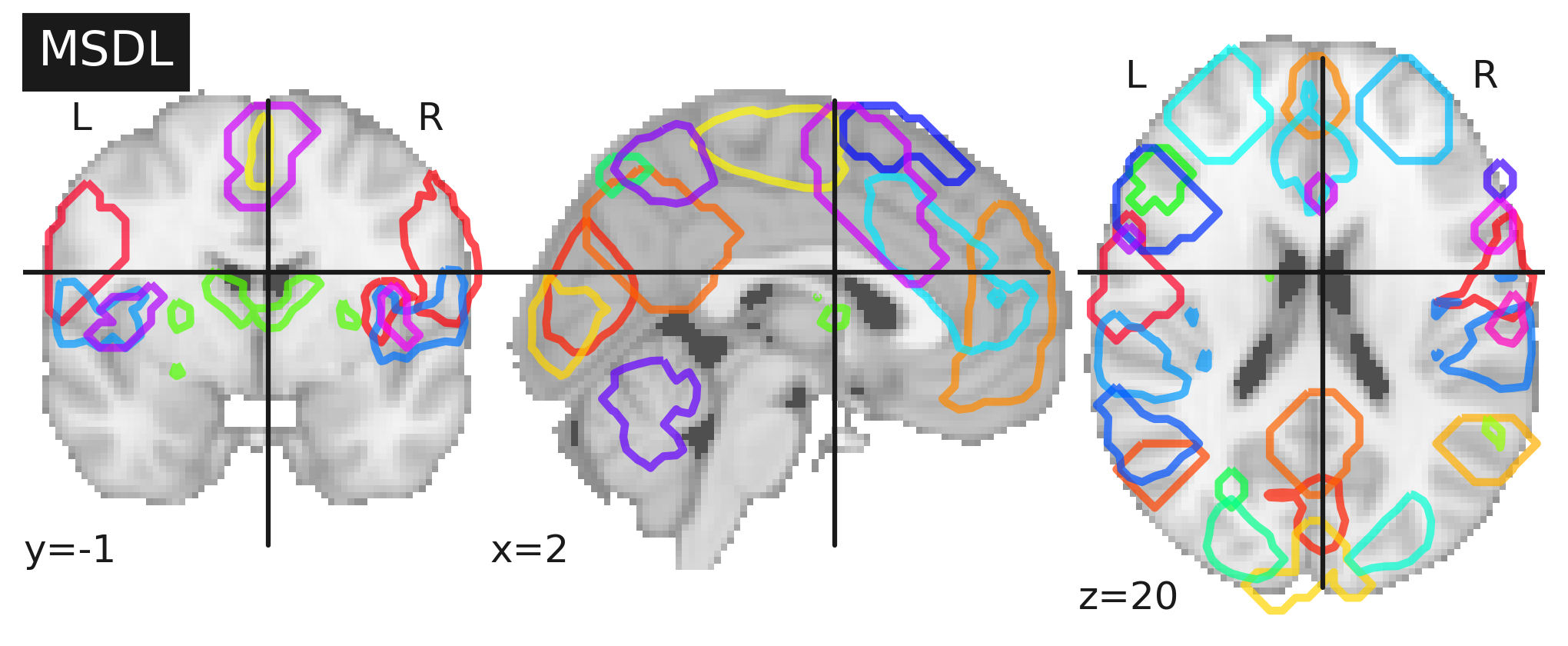


Figure S1: Parcellation with 39 parcels and based on a multi-subject dictionary learning process (MSDL). The parcels appear to be overlapping spatially due to the projections used for plotting.
